# Calling SNPs without a reference sequence

**DOI:** 10.1186/1471-2105-11-130

**Published:** 2010-03-15

**Authors:** Aakrosh Ratan, Yu Zhang, Vanessa M Hayes, Stephan C Schuster, Webb Miller

**Affiliations:** 1 Center for Comparative Genomics and Bioinformatics, Pennsylvania State University, USA; 2 Children's Cancer Institute Australia for Medical Research, University of New South Wales, Randwick, Australia

## Abstract

**Background:**

The most common application for the next-generation sequencing technologies is resequencing, where short reads from the genome of an individual are aligned to a reference genome sequence for the same species. These mappings can then be used to identify genetic differences among individuals in a population, and perhaps ultimately to explain phenotypic variation. Many algorithms capable of aligning short reads to the reference, and determining differences between them have been reported. Much less has been reported on how to use these technologies to determine genetic differences among individuals of a species for which a reference sequence is not available, which drastically limits the number of species that can easily benefit from these new technologies.

**Results:**

We describe a computational pipeline, called DIAL (De novo Identification of Alleles), for identifying single-base substitutions between two closely related genomes without the help of a reference genome. The method works even when the depth of coverage is insufficient for de novo assembly, and it can be extended to determine small insertions/deletions. We evaluate the software's effectiveness using published Roche/454 sequence data from the genome of Dr. James Watson (to detect heterozygous positions) and recent Illumina data from orangutan, in each case comparing our results to those from computational analysis that uses a reference genome assembly. We also illustrate the use of DIAL to identify nucleotide differences among transcriptome sequences.

**Conclusions:**

DIAL can be used for identification of nucleotide differences in species for which no reference sequence is available. Our main motivation is to use this tool to survey the genetic diversity of endangered species as the identified sequence differences can be used to design genotyping arrays to assist in the species' management. The DIAL source code is freely available at http://www.bx.psu.edu/miller_lab/.

## Background

Next-generation sequencing technologies have revolutionized genomics, leading to a tremendous increase in the amount of available sequence data, while bringing down the cost per base. However, de novo assembling of mammalian genomes using these short reads has met with limited success [1], despite recent strides in assembling smaller microbial genomes [1-3]. The market for these short-read technologies has largely been driven by resequencing efforts, where reads are mapped to a genome sequence that was typically assembled using some other sequencing technology such as Sanger sequencing. The deduced genomic differences are then studied to characterize and understand the genetic diversity among individuals of the species. The last few years have seen tremendous progress in the development of algorithms and software to map short reads onto a reference sequence and to identify differences between the individual and the reference [4-6].

Much less has been published about identifying genomic differences in species where an assembled reference sequence is lacking. We describe our computational pipeline, called DIAL, for deducing genetic differences within a target species without the help of a reference genome. The method works even when the depth of coverage is insufficient for de novo assembly. It is also designed to work with sequences from multiple individuals, a situation that can pose difficulties for de novo assembly. We evaluate the performance of DIAL on several extensive datasets.

The directions taken in this study were shaped by our immediate needs, which were to identify a pre-determined number of single-base differences to design a custom genotyping array using only as much sequence data as necessary. With current array technology, at most a few thousand differences can be assayed at an affordable cost (a few tens of thousands of dollars), so our main interest here lies in knowing how to identify that many differences with a low false-positive rate, and in understanding the main sources of error at low levels of sequence coverage.

## Implementation

**Figure 1:**
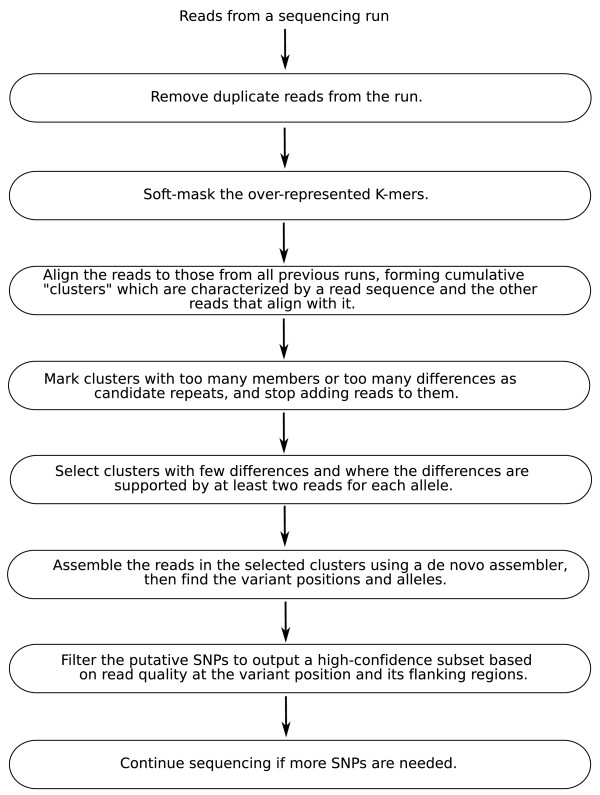
**Outline of the DIAL pipeline to call SNPs**.

DIAL is implemented as a pipeline where the output from one stage serves as the input to the next stage. SNPs (Single Nucleotide Polymorphisms) are called every time a new set of sequences is added. A set can be the yield from a lane of Illumina sequence, a quadrant of 454 sequence, or any subset as decided by the user. Once the number of called SNPs is adequate for the desired application, we can stop sequencing. The general flow of the pipeline can be divided into several steps, as summarized in Figure 1 and described in greater detail below.

### Masking of repetitive sequences

Duplicate reads can arise from PCR duplicates, sequencing artifacts such as poly-A and poly-N reads, noise in cluster detection, and from genomic DNA shearing at the same location in different molecules. These clones can lead to incorrect coverage assessments and erroneous SNPs. We collapse all such clones of a read into a single sequence by using a simple hash function. We identify reads with the same MD5 checksums [7] and if they have identical bases, then we replace them with a single sequence. Interestingly, we found that the Watson Roche/454 sequence dataset had an average of 0.2% duplicate reads in a single set, whereas the observed number for the Illumina reads was higher at about 1.2%.

We then feed the non-duplicate reads to another module, which is based on WindowMasker [8].  WindowMasker uses linear-time scans of a genome to identify repetitive sequences, and has been proven effective in cases where much of the sequence is in draft form. We modified WindowMasker's algorithm slightly so it could be used in conjunction with reads from sequencing runs. We soft-mask probable repeats (i.e., over-represented *K*-mers) by using lower-case letters for nucleotides, so that alignment anchors are not chosen in those regions. We achieve this in three sequential passes, as described below.

1. We use the first pass to calculate appropriate parameters and cutoff values to be used by the module. We define *K*, the size of the *K*-mers to be used, as the largest integer such that (*N*/4^*K*^) > 5, where *N *is the total sequence yield from the current input set. We calculate and store the frequency of each *K*-mer, *freq*(*S*), in a splayhash for efficient access, then sort the *K*-mer frequencies and store the results in a temporary array *F*. If *C *is the total number of unique *K*-mers from the sequences in the set, then cutoffs *T*_*high*_, *T*_*threshold*_, *T*_*extend *_and *T*_*low *_are calculated as *F *[*int*(0.998 * *C*)] + 1, *F*[*int*(0.995 * *C*)] + 1, *F*[*int*(0.99 * *C*)] + 1 and *F*[*int*(0.9 * *C*)] + 1, respectively. We compute a score for each *K*-mer to mitigate the effects of a few *K*-mers with very high frequencies and to avoid keeping track of counts for many infrequently occurring *K*-mers. The score is calculated as:

2. We use the second linear scan to divide each sequence into a set of overlapping windows. Each window contains 5 *K*-mers, and we calculate the score of each window by averaging the scores of the *K*-mers in it. Adjacent windows share 4 *K*-mers, and we mask the contents of a window if it has a score greater than *T*_*threshold*_. We also mask the interval between two masked windows, if the score of each of the windows in it exceeds *T*_*extend*_.

3. The last pass of the masker looks for local repeats. We calculate the frequency distribution of the *K*-mers within a read, and if any of the frequencies exceed *T*_*extend*_, then we mask the whole read. This is particularly effective in reducing the time required to calculate overlaps between read sequences. This pass also unmasks any masked segment less than 40 bp long; the motive here is to unmask the high-frequency *K*-mers in non-repeat sequences.

### Calculation of overlaps

We align the masked sequences from the current input set against all of the masked reads from the sets processed earlier, using a "seed and extend" strategy so that most of the spurious comparisons are discarded at the outset. We use the LASTZ aligner [9], which is a drop-in replacement for BLASTZ [10]. The appropriate scoring matrix for LASTZ varies depending on the error model of the sequencing technology used. For sequences generated with the 454 technology [11], we set the match score to 1 and the mismatch score to 3, along with gap open and gap extend penalties of 1 and 3, respectively. We require a perfect match of at least 40 bp between two reads for them to be considered for gapped extension. We find the endpoints of the alignment by terminating the gapped extension whenever its score falls 10 points below the maximum score observed so far for that alignment path. Two reads are called "aligned" if they have an overlapping segment of at least 100 bp with an identity of 96% or more.

We modified this score set for use with the shorter 75 bp Illumina reads; the gap open and gap extend penalties are both set to 10, while the match score is 1 and the mismatch score is 10. We find the endpoints of the alignment when the score falls 20 points below the maximum score observed on that path. Two reads are called as "aligned" if they have an overlapping segment of 50 bp with an identity of 96% or greater.

For every read in the input, we store its header and sequence along with the names and observed differences of all the other reads that align to it, in a data structure we term as a "cluster". These clusters are cumulative over the sequencing runs. Each new sequence set is initialized with its own set of clusters, in one-to-one correspondence with the reads. The set of masked sequences is then aligned to all of the masked reads from the previous sets, growing the clusters corresponding to each of two reads if the two reads align. We mark the clusters as "candidate repeats" if they exceed thresholds for the number of members (aligning reads) or the number of observed differences. This is done to prevent a combinatorial explosion that would occur if we stored all the details of all the clusters. We stop adding reads to the cluster once it is marked as a candidate repeat, even though its original corresponding read can still be added to other clusters if it aligns with their sequences. The only exception to this filter is in the case of transcriptome datasets, as transcripts have variable abundance depending on their expression in the sample.

One of the inputs to DIAL is the expected length of the target genome. This information is used to calculate the threshold for identifying a pileup of reads. We normally set it to the expected size of the closest available genome sequence if the expected size of the target genome is not known. If the average depth-coverage is *C*, the coverage *k *at each base of the genome approximately follows the Poisson distribution

DIAL calculates a value *C*_*threshold *_that equals the 99.5^*th *^percentile of the cumulative distribution function for the coverage depth. When a sequence set is added and the number of members in a cluster exceeds *C*_*threshold*_, then the cluster is marked as a candidate repeat. A cluster is also marked as a candidate repeat if the number of differences in it exceeds a limit *D*_*threshold*_. For the reported datasets, this threshold was set to 10.

### Determination of SNPs

We can call SNPs after the overlaps from two input sets are calculated. The determination takes place in several steps:

1. First, we filter the clusters to consider only those which have substitution differences supported by at least two reads for each allele (variant nucleotide at the location of a SNP). Such clusters with more than 3 substitution differences, or with two or more such differences that are within 50 bp of each other, are ignored. If the input to the pipeline consisted of sequences from more than one individual, then we also filter to keep only clusters that have at least two reads from each of at least two individuals. These filters are restrictive and increase the false negative rate of SNP discovery. However we find that they also help in reducing the false positive rate, especially at low coverage. This step also ignores clusters which were marked as "candidate repeats"; they are not considered in the SNP calling stage.

2. We then iterate through the filtered clusters and select those containing only member reads that have not been observed in any of the clusters selected before it. This ensures that a read appears in at most one SNP call.

3. We also ignore clusters whose sequences align to any of the completely masked sequences in the earlier steps. This includes reads that have local repeats as determined earlier and reads that had less than 40 unmasked bp. For this we use the same LASTZ parameters and score sets as we did for calculating the overlaps between the reads. This step ameliorates any local frequency bias in a particular input set. We also remove reads whose clusters have been marked as candidate repeats from the member lists of all other clusters.

4. Next we create "micro-assemblies", i.e., de novo assemblies for each selected cluster. For the 454 technology we extract the reads corresponding to the members of a cluster from the original SFF file to create an input file for Newbler, which is part of the software package distributed by Roche/454 with their sequencing machines. We use Velvet [1] for assembling the Illumina short reads. A different assembler can be specified, as long as it works with the particular sequencing technology and generates output in the ACE file format [12].

5. We then read the ACE file output and deduce positions in the assembly where two different alleles are observed with support from at least two reads for each allele.

6. A final, optional step filters the candidate locations to find SNPs which are suitable for genotyping. The filters involve a combination of factors, including the number of variants and reads found in the assembled contig, the distance of the candidate SNP from the ends of the contig and the quality scores for the reads. Another optional filter disallows SNPs in homopolymer regions. In the reported datasets (generated by 454 and Illumina) the following requirements were imposed:

(a) Only one SNP is found in a micro-assembled sequence.

(b) The SNP has at least 50 bp on both sides in the assembled sequence for the 454 datasets, and at least 40 bp on both sides for the Illumina reads.

(c) The minimum quality score for the reads at the site is 20. Reads supporting an allele with a lower quality score are not counted.

(d) The SNP is not part of a homopolymer of length greater than 4 bp in a window of length 13 bp centered on the SNP.

## Results and Discussion

### An overview of the computational problem

Our strategy is to observe the fragments of DNA sequence data produced by a sequencing machine and identify pairs of fragments that overlap, but differ in one or several of the overlapped positions. When the total number of identified variant positions reaches a desired level (depending on the application at hand), sequencing can stop. If the fragments are all from the genome of one individual, then we are looking for heterozygous positions (i.e., different nucleotides from each parent), whereas with sequence fragments from more than one individual, we can also find positions where every individual is homozygous (identical maternal and paternal nucleotides), but at least two individuals differ. The differences we seek are typically single-nucleotide substitutions that occur within a species or population of interest. We call each position at which different nucleotides appear among the individuals being sequenced a "SNP", short for Single Nucleotide Polymorphism. Each variant nucleotide that appears at a SNP we call an "allele"; for this project we make no requirements concerning the frequency of an allele in the population, other than that it occurs in our samples, and we assume there are precisely two alleles at each SNP. Our approach can also detect insertions/deletions of one or several nucleotides, but these are of less interest to us because high-throughput genotyping methods (i.e., techniques to efficiently assess which variants are possessed by each of many individuals) focus on nucleotide substitutions. Here we describe two difficulties with our strategy, explain how we address them, and analyze their effects on our results.

The first difficulty is dealing with sequencing errors, which can masquerade as SNPs. The actual frequency of SNPs depends on the genetic diversity of the species being sequenced. For instance, the frequency of SNPs between two humans averages around one per 1,000 positions, but in some species SNPs are much less frequent. In any case, the rate of errors in individual fragments produced by the sequencing instrument (regardless of the brand) is probably much higher than the rate of SNPs. This means that when we see a nucleotide difference between two overlapping reads, the odds are that it is a sequencing error rather than a true SNP. A cornerstone of our approach is to require that each allele of a putative SNP be observed at least twice, which we believe is significantly more likely to indicate an actual difference than a repeated sequencing error. (Think of sequencing errors as being approximately independent and having a 1% frequency, so a repeated error will strike at 1 in 10,000 positions, compared to, say, 1 SNP per 1,000 positions.) This requires the genomic position to appear in at least two fragments with one allele and two fragments with the other, and raises the following question: Given *λ*-fold genome coverage in random fragments of an individual genome (i.e., the fragments' total length is *λ *times the size of the genome), what fraction of the genome positions will be contained in two or more fragments from each parent? Or similarly when looking for homozygous differences between two individuals that are each sequenced to depth *λ*/2? In theory, the answer is *z*^2^, where *z *= 1 - *e*^-*λ*/2^(1 + *λ*/2). (See the next section for a derivation.) For instance, given 1-fold coverage of an individual, the expected genome fraction where heterozygous positions can be identified is 0.0081. Thus, if a genome has 1 million heterozygous positions, we expect around 8100 of them to be detectable with 1× sequence coverage if no other issues are considered. Under the same conditions, 2× coverage should put 70,000 SNPs within reach of our method. For a genome with fewer heterozygous positions, our potential yield is proportionately less, e.g., 810 SNPs from 1× coverage of a genome with 100,000 heterozygous positions. Typically, we will not know the SNP density in the sequenced individuals when we start a project; we are more likely to know the number of SNPs that can be accommodated on a genotyping array that is within our budget.

The second difficulty is that overlapping reads differing at one position may not be from the same part of the genome; instead they may be from the two copies of a recent duplication that have accumulated a mutational difference. In that case, a detected difference is of no use for a population-genetic analysis because every individual genome of that species may contain both variants and thus falsely appear to be heterozygous at a single location in a genotyping assay. Hence we consider a SNP prediction to be in error if it turns out to be caused by our failure to distinguish copies of a repetitive region. Our main defense against this possibility is to limit the number of sequenced fragments that contain a given SNP. If a genomic interval has hundreds of nearly identical copies throughout the genome (which can easily happen with families of interspersed repeat elements, such as Alus in primate genomes), then even with only 0.5× coverage we will see a pile-up of overlapping reads, which can be easily rejected. The problem comes from regions that have only a few nearly identical copies in the genome. To give a sense of the frequency of these low-copy repeats in one particular species, consider NCBI Build 36 of the human reference genome. There, 2.16% of the bases are in a repeated region (defined here as at least 97% identity over 300 bases) with precisely 2 copies; for 3 or 4 copies the fractions are 0.72% and 0.43%, respectively. It is not obvious how to extrapolate that estimation to the parts of the human genome not represented in the reference assembly (but which may be represented in next-generation sequenced data), or to another species. However, success of our method clearly requires that the fraction of the genome contained in such high-identity, low-copy-number repeated regions be very small, particularly since the fraction of nucleotides in a duplicated region that differ among the copies is likely to exceed the fraction of positions in single-copy regions that are SNPs.

These two difficulties lead to competing constraints on our strategy; we need to thread our way between not having enough overlapping reads and having too many. Moreover, this threading must be done in the dark, because we typically will not know all of the critical parameters, including the number of actual SNPs among the individuals being sequenced and the frequency of recent genomic repeats in the target species. However, we can obtain guidance by using theoretical models that we fit to observed data. For instance, one decision that needs to be made is the upper limit, call it *x*, on the number of reads that appear to contain a SNP. For identifying heterozygous SNPs in a single genome, the fraction that can in theory be identified when requiring two observations of each allele and at most *x *overlapping reads, given *λ*-fold coverage of the genome, is:

For intervals that appear precisely twice in the genome (albeit with a small number of differences), the fraction of differences that will be mis-identified as SNPs is approximately *P *(2*λ*). (These formulas are special cases of more general results derived in the next section.) If we assume there are 800,000 true SNPs in single-copy regions, 30,000 differences in duplicated regions, and 2.3 copies of a duplicated region on average, then Table 1 shows the numbers of correct positions and the relative number of copy-induced false positives; these numbers roughly reflect data observed for humans.

**Table 1:** Numbers of heterozygous positions correctly identified, and numbers of false-positive predictions from low-copy-number repeats, expressed as a percentage of the identified SNPs.

*λ*\*x*	4	5	6	7	8
0.5	473/54.8%	552/65.0%	561/68.3%	561/69.0%	561/69.1%
1.0	4,598/28.6%	6,131/37.9%	6,450/43.5%	6,501/45.9%	6,508/46.7%
1.5	14,119/14.9%	21,179/21.4%	23,386/26.8%	23,915/30.3%	24,021/32.0%
2.0	27,067/7.8%	45,111/11.8%	52,630/16.0%	55,036/19.5%	55,675/21.8%
2.5	40,080/4.1%	73,481/6.5%	90,877/9.4%	97,835/12.2%	100,145/14.6%

The values are given as a function of fold coverage (*λ*, row labels) and the upper bound on the number of overlapping reads (*x*, column labels). For instance, at *λ *= 1.0 and *x *= 5, there are 6,131 correct SNP calls and 37.9% as many duplication-induced erroneous ones. This is a theoretical analysis based on informally fitting a model (see text) to data from the genome of Dr. James Watson.

This model shows the following general trends. The main trend in the percentages is a difference among the rows. Namely, at 0.5× coverage, the number of incorrect calls caused by low-copy repeats is roughly 60% of the number of correct calls, but as the coverage increases, that percentage drops significantly, reaching around 10% at 2.5× coverage. A less dramatic but still noteworthy trend is seen within rows: at 0.5× coverage, allowing more than *x *= 5 overlapping reads makes little difference, while at 2.5× coverage, setting *x *= 6 produces substantially more correct SNP calls, but with a substantially higher false-positive rate. (For even higher levels of coverage, higher values of *x *are appropriate, and DIAL adjusts automatically; see the Implementation section.) The specific values in the table depend heavily on the assumed numbers of SNPs and of differences within low-copy repeats, but similar trends are observed over a wide range of conditions and provide insight into the problem at hand.

Our goal is to perform only as much sequencing as is needed to produce a desired number of SNP predictions with an acceptable false-positive rate. In practice, we impose additional restrictions on our SNP predictions, as described above in the Implementation section, but the two main conditions are that we see each putative allele of a SNP at least twice, and that there not be too many reads that appear to overlap the SNP. The additional conditions, such as high quality scores and lack of other SNP predictions in the assembled region around the SNP, are intended to decrease the fraction of erroneous SNP calls and to provide predictions that are appropriate as input for certain experimental protocols (such as PCR amplification of the position). However, the extra restrictions unavoidably reduce the number of correct SNP calls.

### SNP detection probabilities

Assume that each position in the genome has equal probability to be covered by a read, and that the reads are independently generated. With small read lengths and sparse SNP distributions over the genome, we ignore the correlation of read coverage for adjacent positions. As a result, the distribution of the number of reads covering each position in the genome can be well approximated by a Poisson distribution, whose mean is the average read coverage per base pair. Denote the genome size by *L*, the read length by *l *(≪ *L*), and the total number of reads by *n*; the read coverage per base pair can then be calculated as *λ *= . The probability of a position in the genome being sequenced at least *x *times is therefore . As a special case, we denote the probability of a position being sequenced at least twice as *f*(*λ*) = 1 - *e*^-*λ*^(1 + *λ*).

Our first rule for calling a SNP is that each allele must be observed at least twice. Assume that we sequenced one or more individuals from the same species or population, with equal read coverage for each individual and the total coverage *λ*. For simplicity, we further assume that each putative SNP is biallelic, and we denote the proportion of each allele over all individuals by *p *and *q*, where *p *+ *q *= 1. For example, if only one individual is sequenced, a SNP must be heterozygous with *p *= *q *= 0.5. We can calculate SNP detection probabilities according to the first rule as *f *(*λp*)*f *(*λq*). As a special case, if we sequence two individuals, we have the following results:

1. If both individuals are heterozygotes at the SNP, or they are homozygotes but with different alleles, the probability of both alleles being sequenced at least twice is .

2. If one individual is a heterozygote and the other is a homozygote at the SNP, the probability of both alleles being sequenced at least twice is .

### SNP calling in the presence of segmental duplications

Let *l*_*t *_denote the total length of all distinct segments that have precisely *t *copies in the genome, e.g., *l*_1 _denotes the total length of segments unique in the genome, and *l*_2 _denotes the total length of (one copy of) all segments that appear twice. The genome size is then given by , and the average read coverage per distinct base pair in the genome is . Here we treat base pairs occurring at the same position in multiple duplicates as a collapsed single base pair, i.e., a column in the multiple alignment of duplicated regions. As a result, the expected read coverage at each collapsed base pair is *λt*. The larger *t *is, the higher the chance that there has been a nucleotide substitution in one copy, which could be mistaken by DIAL for a putative "SNP". The term "SNP" is quoted because it may correspond to mutations occurring in different copies of a duplicated region. Our goal is to call SNPs within unique regions in the genome and discard all "SNPs" in duplicated regions (even though some may be real). Without a reference genome, however, we do not know whether a segment is duplicated or not. We therefore select an upper bound for the observed read coverage per base pair, beyond which the corresponding "SNP" will be treated as a duplication difference and discarded. The choice of the upper bound for read coverage can be used to strike a balance between sensitivity and specificity. Suppose that we only call a SNP if (1) both alleles are observed at least twice; and (2) the number of reads covering the "SNP" is at most *x *(≥4). Within unique regions (we abuse notation by calling those regions *l*_1_), the calling probability of each SNP can be calculated as:

The calling probability of a "SNP" in duplicated regions can be similarly calculated with *λ *replaced by *λt*, i.e., *Pr*(call a "SNP" in *l*_*t*_) = *P *(*λt*).

To assign the values of allele proportions *p *and *q *in unique regions, we may let *p *= *q *= 0.5, which is exact if only one individual is sequenced. For regions with *t *copies, the frequency of duplication differences is typically much higher than that of SNPs, and thus when *t *= 2, we will have *p *= *q *= 0.5 most of the time. When *t *> 2, without additional information, we may assume a star tree topology for the evolution of duplicated regions, and thus we can let *p *= 1 -  and *q *= .

Given the above equation, we can define and calculate the sensitivity of our SNP calling (with respect to our stated goal above) as:

Assume that the average density of "SNPs" in a *t*-copy region is *p*_*t*_, and thus the expected number called in all *t*-copy regions is *N*(*t*) = *p*_*t*_*l*_*t*_*P*(*λt*). The specificity of our SNP calling can therefore be defined and calculated as:

Among all of the calls, the proportion of correct SNPs (proportion of true positives) is approximately:

### Watson genome dataset

The genome of Dr. James Watson was the first full genome to be sequenced using a next-generation sequencing technology [13]. Several groups subsequently computed single-nucleotide differences from the human reference sequence, including the inferred heterozygous positions that we used to evaluate the accuracy of our SNP-calling method. Fortunately, we have an extremely accurate assembly of a human reference genome sequence, making it possible to reliably determine which of the positions lie in near-identical, low-copy-number repeats, which are the bane of our strategy for calling SNPs without a reference (at least at low levels of sequence coverage).

We proceeded as follows. Of the 106.5 million high-quality reads that were generated for the Watson project, 76.6 million are available in the Short Read Archive at NCBI. The available reads total 18,952,140,632 bases and provide approximately 6.15-fold coverage of the genome. We downloaded the corresponding SFF files (low-level representations of the sequence data produced by Roche/454 sequencing machines, which include information used by our DIAL pipeline to improve SNP-calling accuracy). DIAL processed this data in about 350 CPU hours on a workstation. Starting at 0.4× coverage, and then at regular increments, DIAL predicted heterozygous positions. Along with each SNP prediction, DIAL reports an interval of assembled sequence that contains the putative variant position. Our subsequent analysis focused on high-confidence SNP calls where no other SNPs were predicted nearby; this was done because our main intended application for DIAL is to make predictions used for subsequent experimental studies, which frequently require variant-free sequences on both sides, e.g., for PCR primers. However, it also helps to eliminate low-copy-number duplicated regions that split more than a few million years ago, because older duplicates will probably have accumulated multiple nucleotide substitutions. (However, subsequent gene-conversion events sometimes erase differences from an older duplication.)

**Figure 2:**
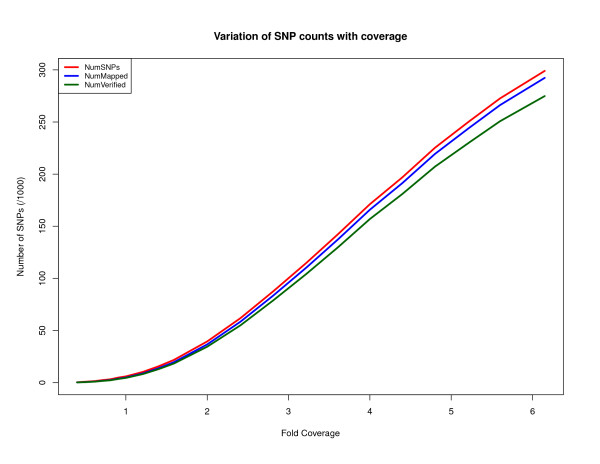
**Variation with coverage (Watson genome dataset)**. A plot showing the variation of several results as a function of sequence coverage. "NumSNPs" is the number of SNPs called by DIAL. "NumMapped" is the number of SNPs and their assembled flanking regions that could be uniquely mapped with greater than 98% identity to NCBI Build 36 of the human genome. "NumVerified" is the number of DIAL SNP calls that were reported for the Watson genome by Cold Spring Harbor Lab, ENSEMBL, or dbSNP.

**Figure 3:**
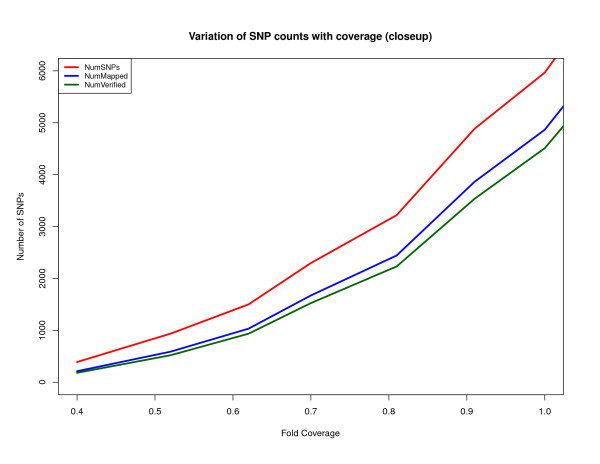
**Magnification of the data in Figure 2 for 1-fold coverage or less**.

Figures 2 and 3 show the number of heterozygous positions that DIAL predicted for the Watson genome at various levels of sequence coverage. For instance, at 1× coverage DIAL identified 5,966 potential SNPs, while at 2× and 6.15× it identified 39,502 and 299,064, respectively.

**Figure 4:**
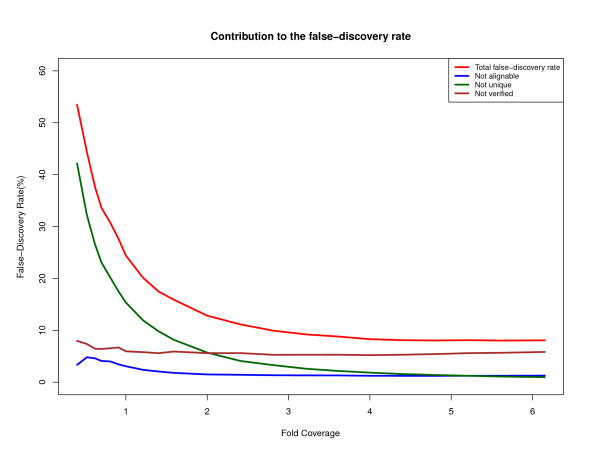
**Rates of erroneous SNP calls associated with various causes (Watson genome dataset)**. "Not verified" means that the SNP was not found in the databases of Watson's heterozygous positions that we consulted. "Not alignable" means that the assembled neighborhood of the SNP did not align to the human reference at 98% identity or higher over at least 200 bp, whereas "Not unique" means that it aligned more than once.

We judged that a heterozygous SNP predicted by our method is correct if (1) the accompanying assembly of flanking sequence aligned to precisely one position in the human reference genome (NCBI Build 36) at appropriately high thresholds (98% identity over 200 bp) and (2) it agreed with any of the Watson heterozygosity calls that we downloaded from three sources (Cold Spring Harbor Laboratory, ENSEMBL, and dbSNP). Figures 2 and 3 plot the total number of SNP calls, the number of SNP regions that appeared to be unique (criterion 1), and the number of these that were verified against the heterozygous positions found by other groups using a reference-mapping approach (criterion 2). Figure 2 indicates that for high genome coverage, the rate of erroneous SNP predictions is around 10%, over half of which appears to be over-prediction in single-copy genomic regions (NumMapped - NumVerified in Figure 2). However, of the 17,300 SNP predictions that DIAL made in single-copy genomic regions at 6.15× coverage but which were not in the other sets of Watson SNP calls, 43% were found in other databases of SNPs, which suggests to us that many and perhaps most of our putative false positives are incomplete heterozygosity calls in the other Watson SNP collections. Figure 4 shows the various sources of error in the false-discovery rate. At 0.5× coverage of the Watson genome, over 50% of DIAL's heterozygosity calls were judged incorrect; the main source is low-copy-number genomic repeats, as discussed above. The coverage and difference filters applied to mark clusters of reads as "candidate repeats" work much better as the coverage increases. A small fraction of the assembled neighborhoods of putative SNPs fail to align to the human reference genome at our requirement of at least 98% identity, though they do align under relaxed conditions.

We also considered the number of false negatives, i.e., heterozygous positions in the Watson genome that seem to meet the conditions we impose but are not identified by DIAL. We estimate that there are about 1 million heterozygous positions that do not lie in a repetitive region or near another heterozygous position, but DIAL finds only 299,064 from 6.15× of sequence data. The reasons for this discrepancy include the difficulty of predicting heterozygous positions with only low-coverage data and the variety of additional conditions (see Implementation) that we require before calling a SNP.

### Orangutan dataset

**Figure 5:**
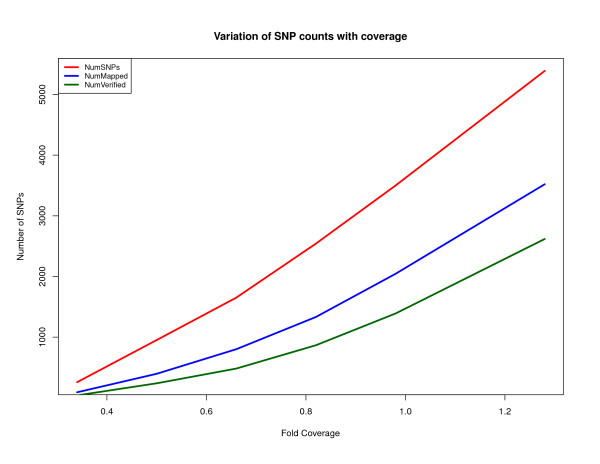
**Variation with coverage (Orangutan dataset)**. A plot showing the variation of several results as a function of sequence coverage. "NumSNPs" is the number of SNPs called by DIAL. "NumMapped" is the number of SNPs and their assembled flanking regions that could be uniquely mapped with greater than 96% identity to the Pongo_abelii-2.0 assembly of the orangutan genome. "NumVerified" is the number of DIAL SNP calls that were verified as present in the read mappings (see text).

Illumina sequencing technology generates short reads only 75 bp long, but the amount of data generated in a single run far exceeds that from the Roche/454 technology. We used a single run of 75 bp reads from a Bornean orangutan *Pongo pygmaeus *which contained 51,923,090 single reads to call heterozygous sites and evaluate our approach on Illumina reads. We adjusted the alignment parameters in the pipeline to make them more amenable to the short reads and were able to identify 5,389 high-confidence SNPs for this dataset. A consequence of the short read lengths is that the assembled contigs for the orangutan reads were about 90 bp, whereas the flanking regions for the Watson genome dataset were around 350 bp. As for the Watson dataset, we plot the number of SNPs called by DIAL and the SNP locations which could be verified in Figure 5.

We had SNP calls from various outside sources for the Watson genome dataset; however no such calls exist for this one. Hence, we verified our calls against a mapping of reads on the orangutan draft assembly (WUSTL version Pongo_abelii-2.0), which we made using MAQ [4]. An allele was deemed to be correct if it was supported by at least one read on the assembly with a mapping quality greater than 0. Even though we make similar number of SNP calls for the orangutan and Watson datasets, the false-discovery rate for these shorter reads is much higher, as deduced by the comparison to the results from MAQ. In order to investigate the reasons for this difference, we mapped a single Illumina run of 75 bp single reads from the genome of a Korean individual [14] to the human assembly. MAQ was able to align 91.9% of the reads, mapping 86% of them uniquely on the human assembly. In contrast, for the orangutan, MAQ was able to align 89% of the short reads, but map only 75.9% of them uniquely on the orangutan draft assembly, much lower than for human assembly. The biggest source of false-positives for this dataset is the number of assembled contigs that could not be uniquely mapped. Many of the contigs map to the unassigned scaffolds (chrUn) along with a mapping on some other chromosome. We believe that our results for this dataset will show a much lower false-positive rate once an improved assembly is available and more data from the scaffolds is assigned to chromosomes.

### Mule deer transcriptome dataset

Compared to generating sequence data from an entire genome, sequencing transcribed DNA (cDNA derived from messenger RNA) has several potential advantages. For a typical mammalian genome, the total length of all gene transcripts is approximately 1% of the genome's length; this means roughly 30 million bases instead of 3 billion. Thus, much less sequence data is required to reach depth-4 coverage for a substantial fraction of the target. And for some applications, the much higher fraction of functional sites is useful. However, there are disadvantages as well. While blood is a typical source of DNA for whole-genome sequencing, the number of genes transcribed in blood cells is rather low. If possible, it may be preferable to sequence DNA from another tissue or combination of tissues. Another complication is that the relative amounts of transcript DNA vary widely among genes, which lowers the efficiency of transcript sequencing (because of redundant data from highly expressed genes), and forces us to remove our constraint on the maximum number of fragments that overlap a SNP.

To experiment with this approach, we applied DIAL to 93 Mbp (422,914 reads) of Roche/454 transcript data obtained from a pooled (i.e., from several individuals) lymph-node cDNA library from mule deer (*Odocoileus hemionus*). Despite the reduced number of genes transcribed in lymph nodes compared to certain other tissues (e.g., brain, testis), we identified 269 high-confidence SNPs, which is sufficient to populate a small genotyping array with SNPs having a good chance of relating to differences in immune-system genes with phenotypic effects. Note that a similar amount of whole-genome DNA would provide only about 0.03-fold coverage, which according to our results discussed above is expected to reveal less than 1 SNP, assuming a total of about 1 million nucleotide differences in the sampled mule deer.

## Conclusions

The application that motivated this project is to use high-throughput sequencing and genotyping methods to help maintain endangered species, for which there is rarely an available reference genome assembly. The use of genome technologies in species conservation efforts is appropriate, given studies that suggest a positive relationship between heterozygosity and breeding success in endangered populations [15,16]. In outline, our strategy is to sequence only as much DNA as is required to design an affordable genotyping array, to then genotype a large number of animals from the species of interest, and finally to use other computational tools [17] to analyze the resulting genotyping data and help to guide reintroduction or captive-breeding programs.

The computational pipeline described above fulfills our needs for calling SNPs without a reference genome. SNPs are called by a completely automatic process that requires only modest amounts of data to make an appropriate number of SNP calls(currently on the order of one thousand). This paper provides a theoretical model of the numbers of correct and false positive calls, as well as experimental data that suggest parameter values for that model. These will help researchers to estimate both the amount of DNA sequence data required to populate a custom-built SNP array and the rate of inevitable incorrect calls caused by low-copy segmental duplications. This information will be useful in practice to balance sequencing costs with SNP-call reliability as the relative costs of sequencing and genotyping fluctuate.

The number of SNPs that one can afford to genotype is typically far less than the total number of common SNPs in the species of interest. Hence, it may be cost-effective to limit sequencing to DNA from specific parts of the genome, so that a given amount of sequence data will produce far deeper coverage. One approach, mentioned above, is to sequence gene transcripts, and thereby target protein-coding regions. Another strategy is to sequence "reduced representation libraries" [18-22], created by size-selecting fragments produced by complete restriction endonuclease digestion, which can target regions distributed throughout the genome. A third approach could be to capture particular intervals by hybridization to a collection of genomic fragments, using either a microarray [23] or a solution-based [24] technique. The methods described in this paper will work for sequence data generated by any of these approaches.

We are applying this strategy to the Tasmanian devil (*Sarcophilus harrisii*), the largest living carnivorous marsupial. The species is currently found in the wild only in the state of Tasmania, Australia, and is under threat of extinction by an aggressive transmittable cancer [25]. The phylogenetically closest available genome sequence is from the laboratory opossum (*Monodelphis domestica*). The two lineages diverged about 100 million years ago, making it impossible to use the opossum sequence as a reference genome for mapping the devil reads. Using Roche/454 machines, we generated low-coverage sequence for two Tasmanian devils from geographically dispersed locations. DIAL then called SNPs, which were used to design a genotyping array. The results of that study will be presented in a separate report.

## Availability and requirements

• Project Name: DIAL

• Availability: http://www.bx.psu.edu/miller_lab/

• Operating system(s): Linux

• Programming language(s): C/Python

• Other requirements: Newbler, Velvet for assembling the flanking regions of the called SNPs

• License: MIT License

• Any restrictions to use by non-academics: None

## Authors' contributions

AR and WM designed the project. YZ performed the probabilistic analysis. AR designed and implemented the DIAL software. VMH and SCS founded the Tasmanian devil genome project, which motivated calling SNPs without a reference. AR and WM wrote the paper with input from the co-authors. All authors read and approved the final manuscript.
